# Improvement of Nanopore sequencing provides access to high quality genomic data for multi-component CRESS-DNA plant viruses

**DOI:** 10.1186/s12985-025-02694-x

**Published:** 2025-03-18

**Authors:** Daniel H. Otron, Denis Filloux, Andy Brousse, Murielle Hoareau, Babbitha Fenelon, Cécile Hoareau, Emmanuel Fernandez, Fidèle Tiendrébéogo, Jean-Michel Lett, Justin S. Pita, Philippe Roumagnac, Pierre Lefeuvre

**Affiliations:** 1 The Central and West African Virus Epidemiology (WAVE) for Food Security Program, Pôle Scientifique et d’Innovation, Université Félix Houphouët-Boigny (UFHB), Abidjan , 22 BP 582 Côte d’Ivoire; 2 CIRAD, UMR PVBMT, St Pierre, La Réunion, F-97410 France; 3 UFR Biosciences, Université Félix Houphouët-Boigny (UFHB), Abidjan , 22 BP 582 Côte d’Ivoire; 4 PHIM Plant Health Institute, University Montpellier, CIRAD, INRAE, Institut Agro, IRD, Montpellier, F- 34398 France; 5 CIRAD, PHIM, Montpellier, F-34398 France; 6 Department of Plant Protection, College of Agriculture, CIRAD, UMR PVBMT, Can Tho University, Can Tho city, Vietnam

**Keywords:** CRESS-DNA viruses, Rolling circle amplification, Nanopore sequencing, Tandem repeat

## Abstract

**Background:**

Faced with the recrudescence of viral CRESS-DNA plant diseases, the availability of efficient and cost-effective tools for routine diagnosis and genomic characterisation is vital. As these viruses possess circular single-strand DNA genomes, they have been routinely characterised using rolling circle amplification (RCA) coupled with Sanger sequencing. However, while providing the basis of our knowledge of the diverse CRESS-DNA viruses, this approach is laboratory-intensive, time-consuming and ultimately ineffective faced with co-infection or viruses with multiple genomic components, two common characteristics of these viruses. Whereas alternatives have proved effective in some applications, there is a strong need for next-generation sequencing methods suitable for small-scale projects that can routinely produce high quality sequences comparable to the gold standard Sanger sequencing.

**Results:**

Here, we present an RCA sequencing diagnostic technique using the latest Oxford Nanopore Technology flongle flow cells. Originally, using the tandem-repeat nature of RCA products, we were able to improve the quality of each viral read and assemble high-quality genomic components. The effectiveness of the method was demonstrated on two plant samples, one infected with the bipartite begomovirus African cassava mosaic virus (ACMV) and the other infected with the nanovirus faba bean necrotic stunt virus (FBNSV), a virus with eight genomic segments. This method allow us to recover all genomic components of both viruses. The assembled genomes of ACMV and FBNSV shared 100% nucleotide identity with those obtained with Sanger sequencing. Additionally, our experiments demonstrated that for similar-sized components, the number of reads was proportional to the segment frequencies measured using qPCR.

**Conclusion:**

In this study, we demonstrated an accessible and effective Nanopore-based method for high-quality genomic characterisation of CRESS-DNA viruses, comparable to Sanger sequencing. Face with of increasing challenges posed by viral CRESS-DNA plant diseases, integrating this approach into routine workflows could pave the way for more proactive responses to viral epidemics.

**Supplementary Information:**

The online version contains supplementary material available at 10.1186/s12985-025-02694-x.

## Background

Plant viruses cause significant losses in agriculture, affecting yield and produce quality. They account for almost 50% of plant diseases worldwide, with economic losses estimated at €30 billion annually [1]. As viral plant diseases continue to emerge and re-emerge globally, there is a need for efficient and cost-effective genomic analysis tools tailored to the small genome size of most plant viruses to diagnose and monitor these diseases. However, viruses are a polyphyletic group of organisms [2] and do not present any canonical gene, such as the 16 S gene in bacteria [3] making it challenging to develop universal methods for virus identification and genomic characterisation. Multiple metagenomic approaches have been developed (see Roossinck et al. [4] for detailed descriptions) and have proved effective in viral detection, but routine full-genome characterisation remain difficult. Instead, methods for specific groups of viruses are required. One of these groups is the Cressdnaviricota phylum which features circular Rep-encoding single-stranded (CRESS)-DNA virus. This group of viruses infects a range of organisms from plants to animals [5]. In particular, this phylum includes the *Begomovirus* genus (Family of *Geminiviridae*) and the *Nanovirus* genus (Family of *Nanoviridae*), both associated with diseases in vegetables (e.g. broad bean and tomato) and staple crops (e.g. cassava) [6–8].

The first genomic revolution for CRESS-DNA viruses was the description in a seminal paper of a cloning protocol using Rolling Circle Amplification (RCA) [9] that did not require any knowledge of the viral genomic sequence (henceforth called the RCA-RFLP procedure). It produces high-molecular-weight, linear, double-stranded DNA, tandem-repeat copies (multimers) of the original viral circular ssDNA input template (monomer). After restriction using a selected single-cut enzyme, unit length fragments are cloned and Sanger sequenced [9]. While the *phi*29 DNA polymerase and its derivative amplify both linear and circular DNA matrices, it is particularly effective for circular DNA, resulting in the enrichment of specific CRESS-DNA viral sequence fragments [10, 11]. The widespread adoption of this method played a key role in revealing the diversity of CRESS-DNA viruses and resulted in the discovery of hidden phylogenetic lineages, filling in the gaps in genomic architecture of these small viruses [12]. Nevertheless, while the RCA-RFLP procedure has been very successful, it remains time-consuming, most notably for multi-component viruses [5] or multiple infections [13]. Indeed, after random amplification and restriction, it is still challenging to clone every single component, especially for low abundance DNA molecules [11]. Nanoviruses genomes can contain up to eight different molecules [7], and simple cloning of components using this methods remains complex. Multiple screenings of a large number of bacterial colonies containing recombinant plasmids are thus frequently required.

To bypass this limitation, more recent protocols have combined the RCA procedure with short (Illumina) and long (Oxford Nanopore Technologies, ONT) read sequencing [11, 14–18]. Whereas Illumina sequencing has hitherto been used for large-scale projects [19], the portability of MinION and its ability to rapidly and accurately generate results make it suitable as a small-scale laboratory tool for routine diagnosis and surveillance of viral diseases [14, 20]. However, when applied to CRESS-DNA viruses and compared to sequences obtained with the method of reference (combining RCA-RFLP, cloning and Sanger sequencing), the final genomic assemblies still contain specific errors associated with Nanopore sequencing [21, 22]. While this method proved successful for the detection and characterisation of viruses from multiple sample types [23], it is not yet fully interchangeable with Sanger sequencing.

Here, we build on (i) improvements in ONT sequencing and on (ii) modifications of the protocol published by Ben Chehida et al. [21] to achieve high quality sequencing of the CRESS-DNA genome. The first improvement was a lower basal error rate using the latest ONT products; whereas modal read accuracy was up to Q16 (i.e. 97.5% raw read accuracy over) with R9.4 chemistry, it improved to up to Q20 (i.e. over 99% raw read accuracy) with the latest R10.4 [24]. Most importantly, the second area of improvements relates to the use of the multimeric nature of RCA products: ONT sequencing routinely produces reads of several dozen kb. We were thus able to sequence full-multimers obtained from RCA and treat each multimer as a technical repeat of the sequencing of the same matrix [25, 26]. From the multimers, it was possible to produce high-quality corrected monomers and assemble full genome sequences that exactly matched (100% identity) to the consensus of Sanger sequence clones.

The protocol was validated using two different sample types. One was a sample of a symptomatic cassava infected with African cassava mosaic virus (*Begomovirus manihotis*, family *Geminiviridae*, ACMV), the other was a common vetch plant experimentally infected with faba bean necrotic stunt virus (*Nanovirus necropumiliviciae*, family *Nanoviridae*, FBNSV). The full genome of ACMV and FBNSV were recovered and were similar to those obtained with Sanger sequencing. Crucially, the two components of the ACMV genome and the eight components of the FBNSV genome were recovered in a single procedure, making this approach even more attractive for multi-component viruses, a common feature in plant-infecting CRESS-DNA viruses. Finally, comparison of the number of sequences obtained for each nanovirus component following Nanopore sequencing and quantification of these components using state-of-the-art processes revealed a highly significant correlation, opening new avenues for the use of the MinION sequencer as a quantitative tool for the study of these and similar viruses.

## Materials and methods

### Sample and DNA extraction

Two plant samples were used in this study. The first consisted of leaves collected from a cassava (*Manihot esculenta*) plant displaying cassava mosaic disease symptoms including mosaic and slight leaf curling. Leaves were collected in a cassava field in Kpada, in the region of Nawa in Côte d’Ivoire (-6.413939° W, 5.769340° N), in 2022 using the protocol described by Doungous et al. [27]. The second sample consisted of leaves from a *Vicia sativa* (common vetch) plant infected with FBNSV. The FBNSV clone was agroinoculated to broad bean (*Vicia faba*), then transmitted to *V. sativa* by aphids (*Aphis craccivora*). The agroinfectious clones of FBNSV were produced using isolates (KC978974-KC978979, KC978981 and KC978988) obtained by Grigoras et al. [28]. Total DNA extractions were performed as per the manufacturer’s instructions using the DNeasy Plant Pro kit (Qiagen, Les Ulis, France) and the DNeasy Plant DNA extraction kit (Qiagen, Les Ulis, France) for the cassava and common vetch samples, respectively. After extraction, the DNA was quantified using Qubit dsDNA BR Assay kit on Qubit 4 (Thermo Fisher Scientific, Illkirch, France).

### Rolling circle amplification (RCA) and minion sequencing

For the cassava DNA extract, rolling circle amplification (RCA) was performed with EquiPhi29 polymerase (Thermo Fisher Scientific, Illkirch, France). Approximately 50 ng of total DNA was mixed with 0.5 µL of 10X EquiPhi29 Reaction Buffer, 1.0 µL of exo-resistant random primers and nuclease-free water in a final volume of 10µL. The mixture was then incubated at 95 °C for 3 min and cooled on ice for 3 min. After cooling, 1.5 µL of 10X EquiPhi29 Reaction Buffer, 0.2 µL of 100 mM DTT, 2 µL of 10 mM dNTP mix (Thermo Fisher Scientific, Illkirch, France), 1 µL of EquiPhi29 DNA polymerase and 9.3 µL of nuclease-free water were added to the mixture. Amplification was performed at 45 °C for 3 h, followed by 10 min at 65 °C for polymerase deactivation. After amplification, RCA products were cleaned using Sera-Mag Select Size Selection beads (GE Healthcare, Buc, France) using a 1:2 ratio (volume RCA products/volume beads) and eluate (volume of 10 µL). As RCA amplification results in branched (in opposition to linear) double stranded DNA, the 10 µL eluate was digested with 1 µL of T7 Endonuclease I (NEB, Evry, France) and 2 µL of 5× buffer in a 10 µL reaction volume at 37 °C for 1 h for debranching. After digestion, the fragments were purified using Sera-Mag Select Size Selection beads in a 1:0.65 ratio (volume sample/volume beads) and eluted with 15 µL of purified water. This beads ratio allow to select fragments above ~ 500pb, preventing the sequencing of small fragments later in the procedure. For the common vetch DNA extract, the rolling circle amplification, T7 Endonuclease digestion and purification were performed as described by Ben Chehida et al. [21]. Sequencing libraries were prepared in accordance with the manufacturer’s instructions using the Native barcoding kit (SQK-NBD114.24) for cassava samples and Ligation sequencing kit (SQK-LSK110) for the common vetch sample. Sequencing was then performed separately using one flongle FLO-FLG001 (R10.4) for each experiment on a MinION device (Mk1B, Oxford Nanopore Technologies) and monitored with MinKNOW 22.035. The cassava sample was sequenced in multiplex with two other samples not described here (use of three barcodes for the run).

### MinION sequencing data analysis

**Fig. 1:**
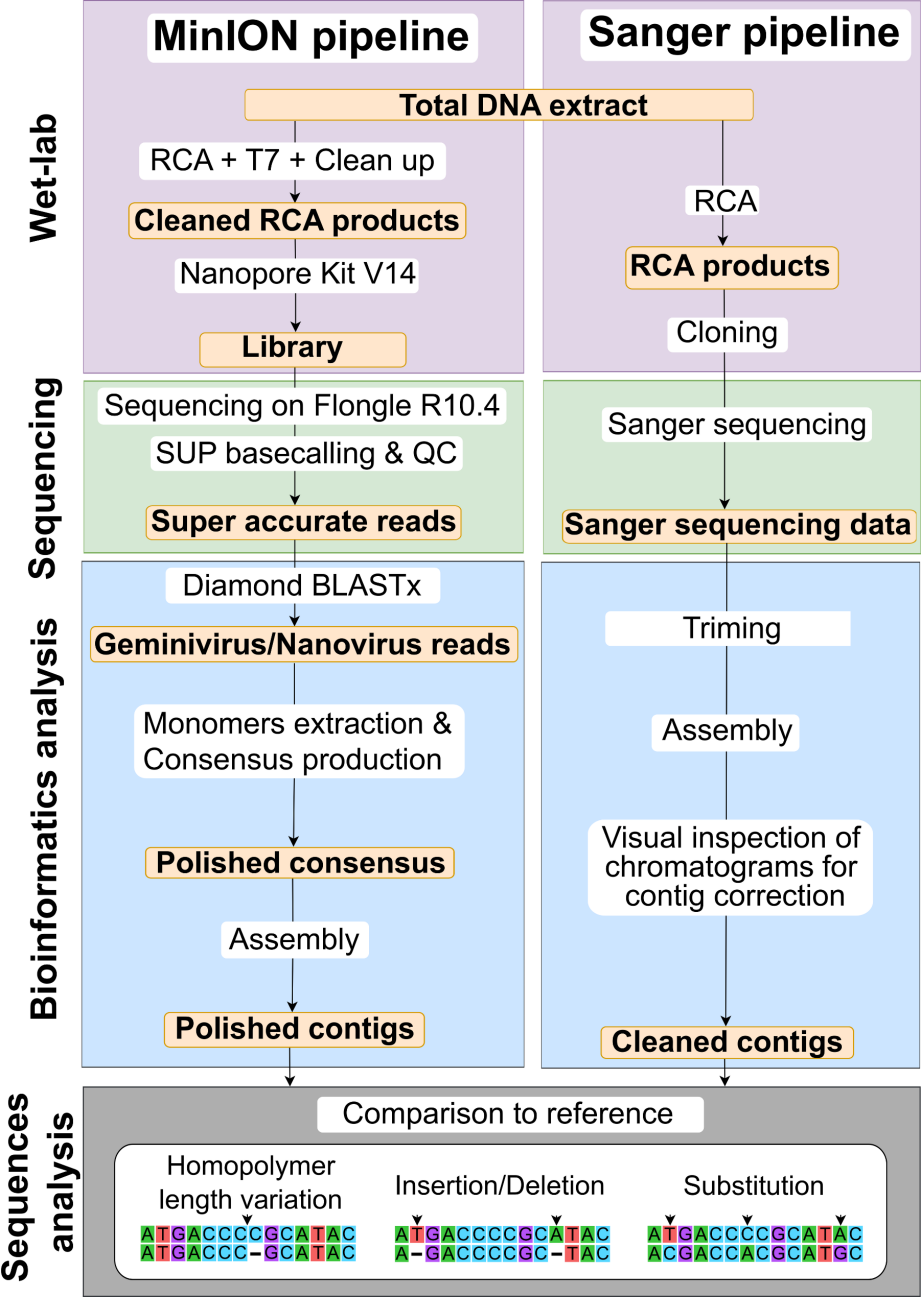
Schematic representation of the pipeline used for comparison of MinION and Sanger sequencing, adapted from Ben Chehida et al. [21]. Wet lab experiments are in purple, sequencing in green, bioinformatics analysis in blue and sequence comparison analysis in grey

After sequencing, raw electric signals were subjected to super accurate basecalling using Guppy v6.5.7 [29] with demultiplexing and adapter removal options. Reads quality was assessed with NanoPlot v1.41.6 (https://github.com/wdecoster/NanoPlot?tab=readme-ov-file) and the reads with mean qualities higher than 7 were kept for downstream analyses. The cleaned reads were subjected to a similarity search using the “blastx” algorithm in Diamond2 [30] against the non-redundant protein database (http://ftp.ncbi.nih.gov/blast/db/FASTA/nr.gz, retrieved from NCBI in January 2024) with an E-value of 0.001 as the cut-off threshold. Reads presenting similarities to CRESS-DNA viruses were then submitted to TideHunter v1.5.4 for the extraction of tandem repeats sequences (TRs). From each read, a draft consensus was generated as described by Gao et al. [25] before polishing with their own TRs using Medaka v1.7.2 (https://github.com/nanoporetech/medaka) to generate tandem repeats consensus (TRCs). The quality of the raw reads, TRs and TRCs was assessed using NanoPlot v1.41.6. TRCs of each DNA component were then assembled separately using Canu v2.3 [31]. When possible, the resulting contigs were manually sliced at the origin of replication and the monomers obtained were assembled with Geneious Prime 2024.0.5 (https://www.geneious.com) (Fig. 1).

### Full genome cloning and Sanger sequencing

The sequences of the eight agroinfectious clones were used for comparison with the MinION assemblies for the common vetch sample. The RCA-RFLP procedure followed by Sanger sequencing was used for the cassava sample. Viral genomes were amplified with rolling-circle amplification using EquiPhi29 as described above. Amplified products were digested with a series of restriction enzymes. The *Aat*II and *Nco*I endonucleases presented with a single cutting site on the DNA-A and DNA-B genomic components, respectively, and were selected for further experiments. Cloning, sequencing and genome assembly were then performed as described in Ben Chehida et al. [21] (Fig. 1).

### Sequence analyses

The sequences obtained from MinION and Sanger sequencing were aligned with MAFFT v7.453 [32] before manual inspection and alignment editing, if needed. The number of mutations between sequences from the clean alignments were counted and classified in substitution, insertion/deletion (INDELs) and homopolymer length variations (HLVs, a subcategory of INDEL; Fig. 1) using custom R scripts. A minimum spanning tree of all genomic sequences obtained in the analysis was constructed using the “spantree” function of the vegan R package [33].

### Quantification of nanovirus DNA

To quantify FBNSV segment accumulation, qPCR was carried out on the common vetch sample. qPCR and the genome formula (GF; i.e. the relative count of every genomic component) determination were performed using the protocol described by Sicard et al. [34]. The genome formula of FBNSV was also determined from Nanopore sequencing data after classifying each TRC sequences as a one of eight genomic component of FBNSV using BLASTn against a database of clone reference. The genome formula obtained using each method were compared using the Pearson correlation test [35].

## Results and discussion

### ACMV and FBNSV Sanger references

Thirty-three clones of ACMV were assembled from Sanger sequencing data from the cassava sample, with sizes ranging from 2,726 to 2,788 nt. Of these, 29 (2,784–2,788 nt) were closely related to the DNA-A component of the Burkinabe isolate of ACMV (LC658347) with nucleotide identity ranging from 98.35 to 98.46%. The remaining four clones (2,726 nt) were most closely related to the DNA-B component of the Ivorian isolate of ACMV (AF259895) with nucleotide identity ranging from 95.52 to 95.63%. The full genome sequences of the ACMV isolates are available on GenBank under the accession numbers PQ261058-PQ261070. For the common vetch sample, the Sanger sequences of the eight component sequences of FBNSV had previously been obtained by Grigoras et al. [28].

### MinION sequencing run data

MinION runs resulted in a total of 21,362 and 542,538 raw reads for the cassava (infected) and common vetch (inoculated) runs, respectively (Table 1). Whereas the yield from the common vetch sample was in line with expectations, the number of reads from the cassava sample was low and indicative of a poor run. Sample quality (such as chemical residue in the sample) or flongle quality are most likely to explain this variation. After barcode trimming, 4,676 reads were assigned to the barcode of the cassava sample, of which 4,545 (97.2% of the raw cassava reads) passed the quality filter. Of the raw common vetch reads 512,363 (94.4%) remained after quality filtering. The average read lengths were 3,910 nt (53–67,684 nt) and 2,158 nt (10–198,306 nt) with median read lengths of 2,381 nt and 1,212 nt for cassava and common vetch samples, respectively. The mean quality were of 13.4 and 15.0 for cassava and common vetch samples, respectively (Table 1; Additional Fig. 1). The observed variation in read numbers and lengths could reflect intrinsic differences in sample types or RCA efficiencies.

Of all the reads that passed quality control and after sequence similarity searches, 1,178 (26.0%) of the cassava sample reads and 139,963 (27.3%) of the common vetch sample reads were assigned to the *Begomovirus* and *Nanovirus* genera, respectively.

**Table 1:** Metrics of the minion sequencing run and assignments of taxonomic Reads

Sample	Total number of reads	Reads passed quality control	Average of read length	N50	Mean quality	Reads of Begomovirus / Nanovirus
Cassava	21,362	20,336 [4,545]	3,910.5 [3,744.6]	7,227 [6,904.0]	13.4 [12.9]	1,355 [1,178]
Common vetch	542,538	521,363	2,158.1	3,745	15.0	139,963

Numbers in brackets indicate the values for the cassava sample described in this study

### Improvement of sequence quality using multimer information

Of the 1,178 reads for begomovirus, 307 (26.1%) represented multimers of at least two copies of DNA genomic components and of the 139,963 reads for nanoviruses, 54,864 (39.2%) represented multimers of at least two copies of DNA genomic components. For the cassava sample, multimeric reads presented 2 to 67 copies of begomovirus DNA components with a mean of 3.9 and a median of 3.0. For the common vetch sample, multimeric reads presented 2 to 65 copies of nanovirus DNA components with a mean of 6.2 and a median of 4.3.

**Fig. 2:**
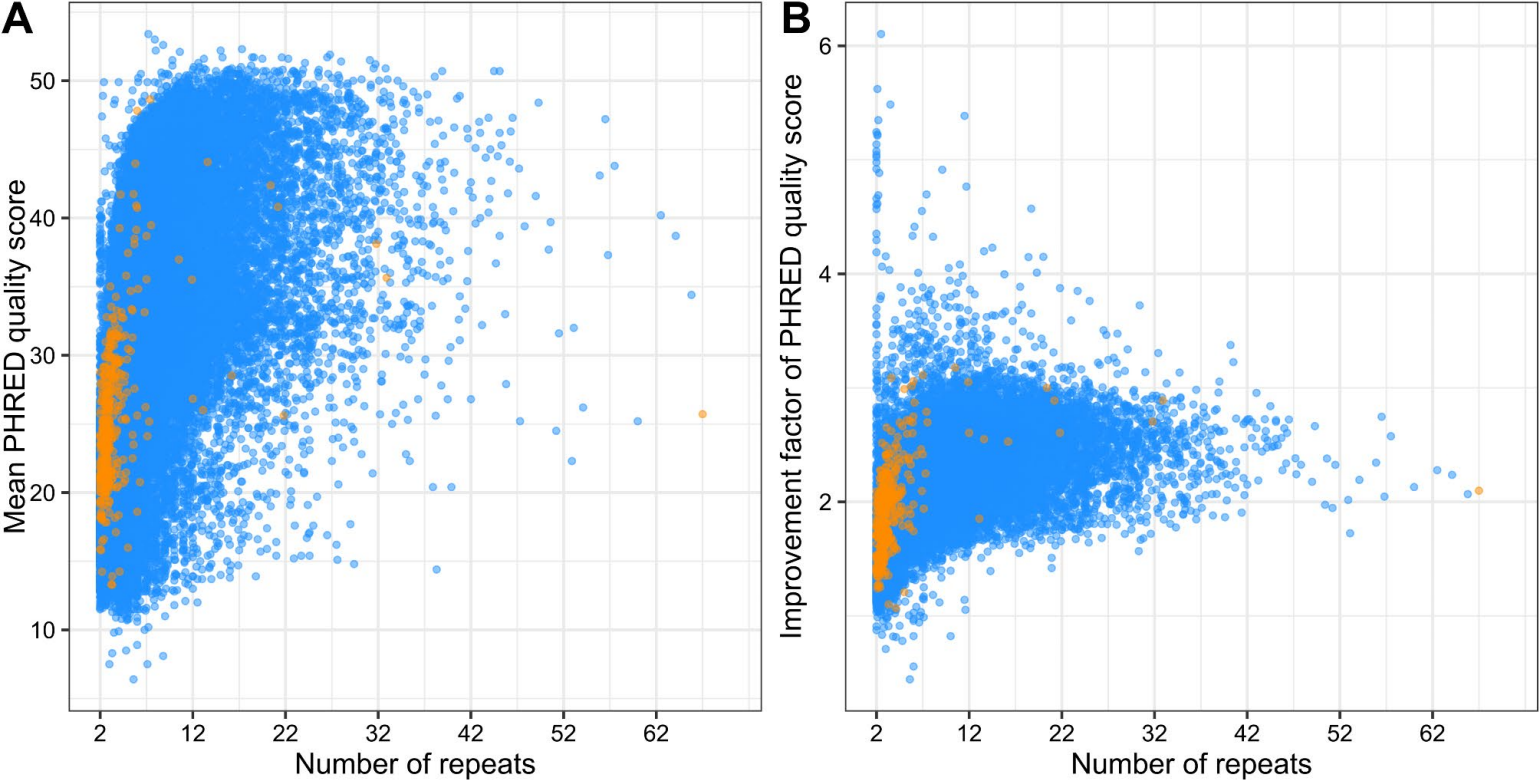
Mean quality scores of the tandem repeat consensus (**A**) and ratios of quality improvement (**B**) in relation to the number of repeats identified in the MinION reads for ACMV (orange dots) and FBNV ssDNA components (blue dots) sequencing run

Tandem repeats (TRs) sequences were then extracted from the reads before generating draft consensus sequences. After polishing, the mean PHRED quality of the Tandem Repeats Consensus (TRC) sequences was of 25.7 (ranging from 13.3 to 48.6) for the begomovirus (mean 25.7). For the nanovirus TRC, mean PHRED quality was of 28.8 (ranging from 6.4 to 53.4; Fig. 2A). This is to be compared with the mean read quality score of 13.4 and 15 that were obtained for the cassava and common vetch after super accurate basecalling but before correction using the multimer information. This represents and increase in accuracy from 94.4 to 99.7% and 96.0–99.9% for the cassava sample and the common vetch sample, respectively. Note that these increases (+ 5.3% and + 3.9%) are higher than the published numbers associated with the transition from the oldest to the newest chemistry (1.5% [24]),. However we cannot disentangle the specific contributions of improved chemistry and improved basecaller, it is also greater than the increase in read quality after basecalling (+ 1.4% accuracy) observed between this work and that of Ben Chehida et al. [21] where chemistry R9.4 was used.

Importantly, our results showed that for the majority of the TRC sequences generated, the mean PHRED quality score increased with the number of TRs (Fig. 2). Comparison of quality scores between TR and TRC sequences showed quality improvements ranging from 1.1 to 3.2 fold (mean 1.9) and from 0.4 to 6.1 fold (mean 1.9) for begomovirus and nanovirus DNA components, respectively (Fig. 2B).

### The identity of TRC sequences compared to Sanger references increases with the number of tandem repeats and the quality score

**Fig. 3:**
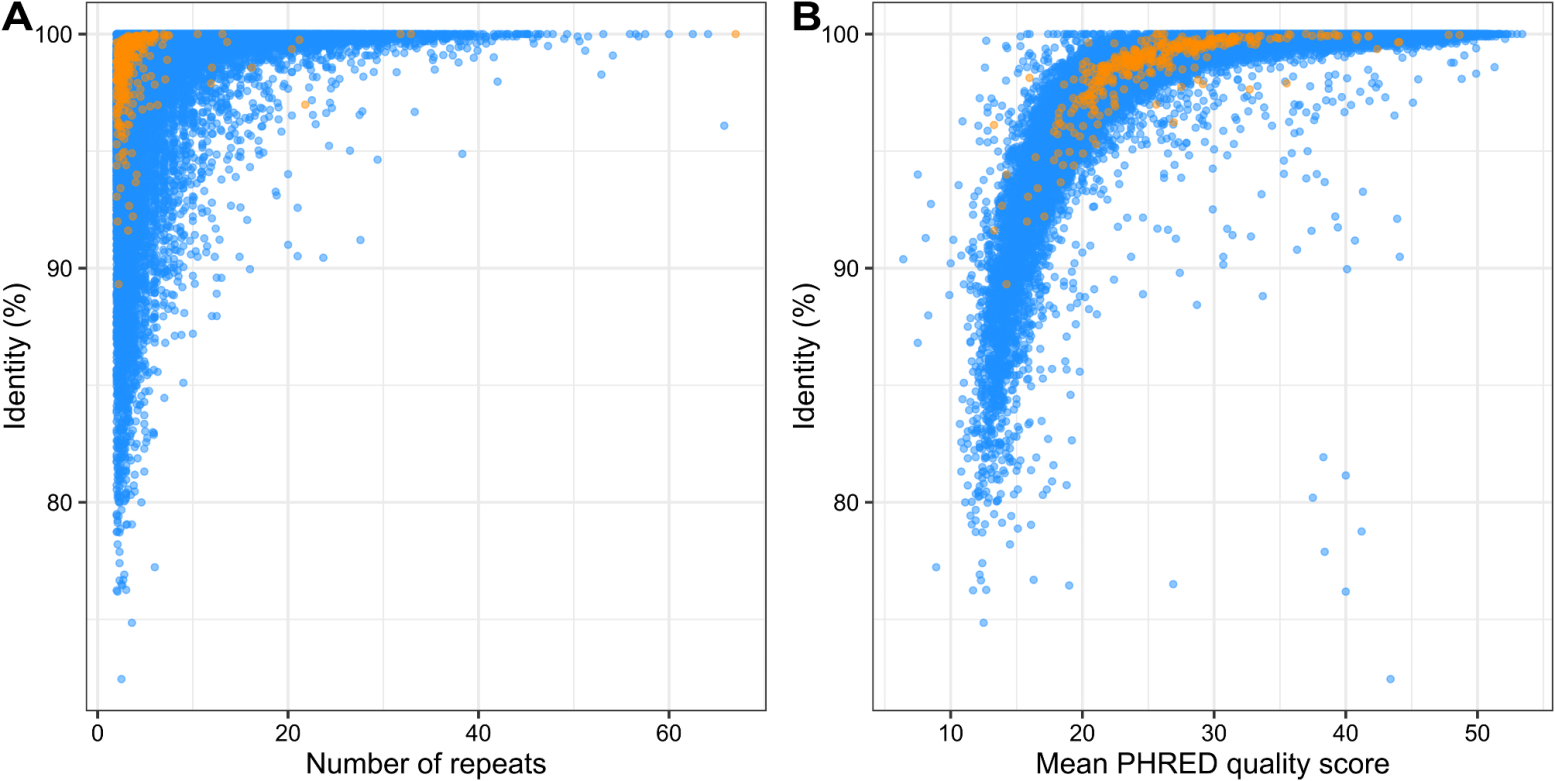
Identity between the tandem repeat consensus and the closest Sanger reference sequence in relation to the number of tandem repeats used in the consensus (**A**) and mean PHRED quality score (**B**) for cassava (orange dots) and common vetch (blue dots)

TRC sequences were then compared to the Sanger viral reference sequences using nucleotide similarities (BLASTn). Noticeable increases in identities of the begomovirus and nanovirus TRC sequences compared with the Sanger reference sequences were observed with increasing tandem repeats (Fig. 3A) and quality scores (Fig. 3B). TRC sequences obtained from three or more repeats had a mean identity of 99.1% compared to the reference whereas TRCs obtained from fewer than three repeats had an identity of 97.2%. The mean quality of TRC sequences obtained from three or more repeats was 31.4 but only of 21.5 for TRC sequences obtained from less than three repeats. Besides confirming the similarities of the begomovirus and nanovirus TRC sequences with the Sanger reference, this indicates that selection for long reads before sequencing or using a threshold of repeat numbers would significantly improve the accuracy of our consensus sequences.

### Sequence comparison reveals a highly accurate minion consensus

**Fig. 4:**
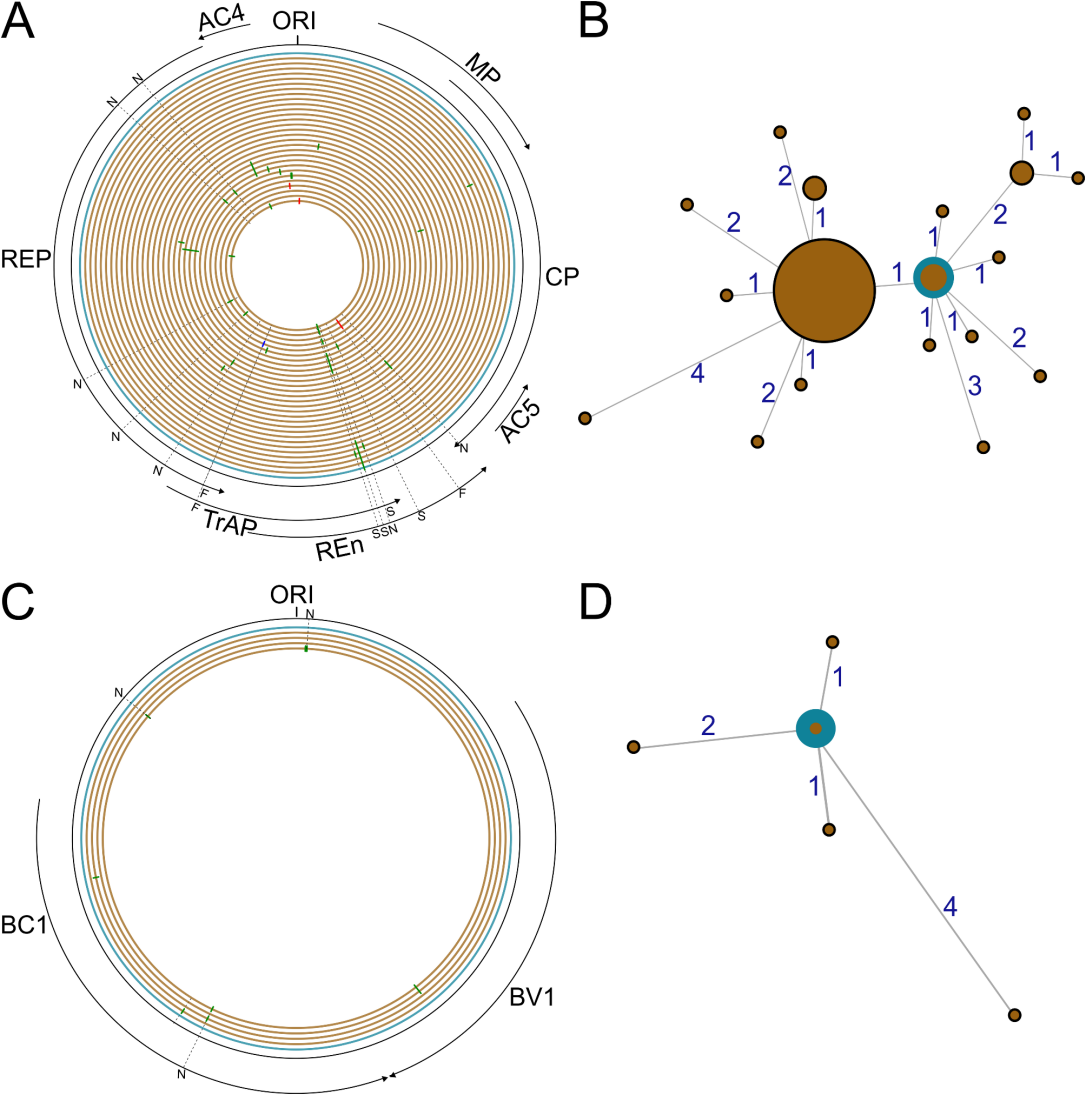
Genomic map showing mutations and their positions (**A** and **C**) and minimum spanning tree (**B** and **D**) for the full DNA-A and DNA-B components of ACMV. Concentric circles represent each complete genome sequence obtained from RCA-RFLP-Sanger (brown) and RCA-MinION (blue) sequencing from the inner circle to the outer circle. Homopolymer length variations (HLV), INDELs and substitutions are represented with red, blue and green ticks, respectively. The N, S and F on the ORFs indicate non-synonymous mutations, mutations inducing a stop codon or mutations inducing a frameshift, respectively. The origin of replication (ORI) is indicated at the top and the ORFs are represented on the outside of the outer circle. The minimum spanning tree showing for DNA-A (**B**) and DNA-B (**D**) the relation between Sanger sequences (brown) and the MinION consensus sequence (blue). Circle sizes are proportional to the number of sequences they represent. Numbers at the edges indicate the number of mutations the edge represents

After assembly of the TRC sequences, two contigs and eight contigs were obtained for the begomovirus and the nanovirus, respectively. The begomovirus contigs corresponded to the full genome sequence of the DNA-A (PQ261071) and DNA-B (PQ261072) components of ACMV. The eight nanovirus contigs corresponded to the full genome sequence of the eight DNA components of FBNSV. The full sequences were then compared to the sequences obtained from Sanger sequencing. The nucleotide differences between these full-length sequences were placed into three categories: HLV (red ticks), INDEL (blue ticks) and substitutions (green ticks) (Fig. 4A and C). Twenty-six mutations were observed in the 29 DNA-A sequences (Fig. 4A and B) and four observed in the four DNA-B sequences (Fig. 4C and D) sequences. All clones were unique. Nine of the 26 mutations detected in the 29 DNA-A sequences were non synonymous or caused frameshifts associated with TrAP and REn truncation, relative to the ACMV reference sequences. Whereas the MinION consensus sequence obtained for the DNA-A component differed from the Sanger consensus by a single mutation, it was identical to two of the Sanger sequences. The Sanger consensus was identical to nine of the 29 Sanger sequences. The mutation observed in the MinION consensus was present in twelve of the Sanger sequences and was associated with a premature stop codon in the REn ORF (Fig. 4A), resulting in a protein length truncation from 134 to 74 amino acids. All other substitutions and HLVs were observed solely when comparing Sanger sequences to each other (Fig. 4A). For the DNA-B, three of the seven mutations observed in the Sanger sequences were non synonymous and one was linked to variations in the BC1 gene (Fig. 4C). INDEL and HLV were not observed in the DNA-B sequences. The MinION consensus was identical to the Sanger consensus (Fig. 4D).

We then analysed TRC sequences to assess to what extent the mutations uncovered through Sanger sequencing of multiple viral clones were captured using the MinION procedure. To that end, we selected TRC sequences with a mean quality equal or superior to 30, resulting in 42 and 13 TRC sequences for the DNA-A and DNA-B components, respectively. Of the 26 mutations that were uncovered in the DNA-A Sanger sequences, four were found in the TRC sequence with frequencies ranging from 1/42 to 27/42. Interestingly, the single mutation distinguishing the MinION and Sanger consensus was found in 27 out of the 42 TRC sequences, demonstrating that it is a circulating mutation. For the DNA-B component, four of the seven mutations found in the Sanger sequences were found in the TRC sequences with frequencies ranging from 2/13 to 7/13. It must be noted, however, that our resolution for DNA-B was lower due to the small number of high-quality TRC sequences for that component. The details of the frequency of each mutation are presented in Additional Table 1. Interestingly, this analysis showed that TRCs may be used to track SNP in sequences. Further analysis would be required to determine how it could be used for a more thorough variant profiling of virus populations.

For the common vetch sample, no RCA-FRLP, cloning and Sanger sequencing was required as the sequences of the eight FBNSV components (DNA-C, DNA-N, DNA-M, DNA-R, DNA-S, DNA-U1, DNA-U2 and DNA-U4) obtained from the TRC sequence assemblies were directly compared to those of the agro-infectious clones used for the experiments. The comparison between the MinION sequences and those used for agroinoculation (Sanger sequences) showed no INDEL, HLV or substitution. Thus, using MinION sequencing, we were able to reconstruct the complete genome of a multipartite virus that was experimentally inoculated.

Whereas the number of reads obtained for FBNSV was relatively high (mean of 17,495 per component and a minimum of 673 reads for the less sequenced component), it was significantly lower for the ACMV. From the 1,007 and 171 reads that were obtained for the DNA-A and DNA-B components respectively, it was nevertheless possible to obtain sequence assembly 100% identical to the Sanger sequence. It could indicate that the level of multiplexing routinely used with the Ben Chehida protocol (twelve sample per flongle [21]), could be employed here. It must however be kept in mind that sequencing yield would largely depend on sample type and quality and sequencing depth would depend on sample complexity and potential imbalances in the number of copies of the different viruses or component in a potential coinfection. Importantly, we have proven that it is possible to recover up to eight components in a single experiment, mimicking a complex co-infection scenario. However, with highly unequal copy numbers for different viruses, a limit would be reached where a virus might fail to assemble in high quality contigs or even be detected. This limitation would potentially be exacerbated with potential RCA biases [36].

### Towards quantitative sequencing

**Fig. 5:**
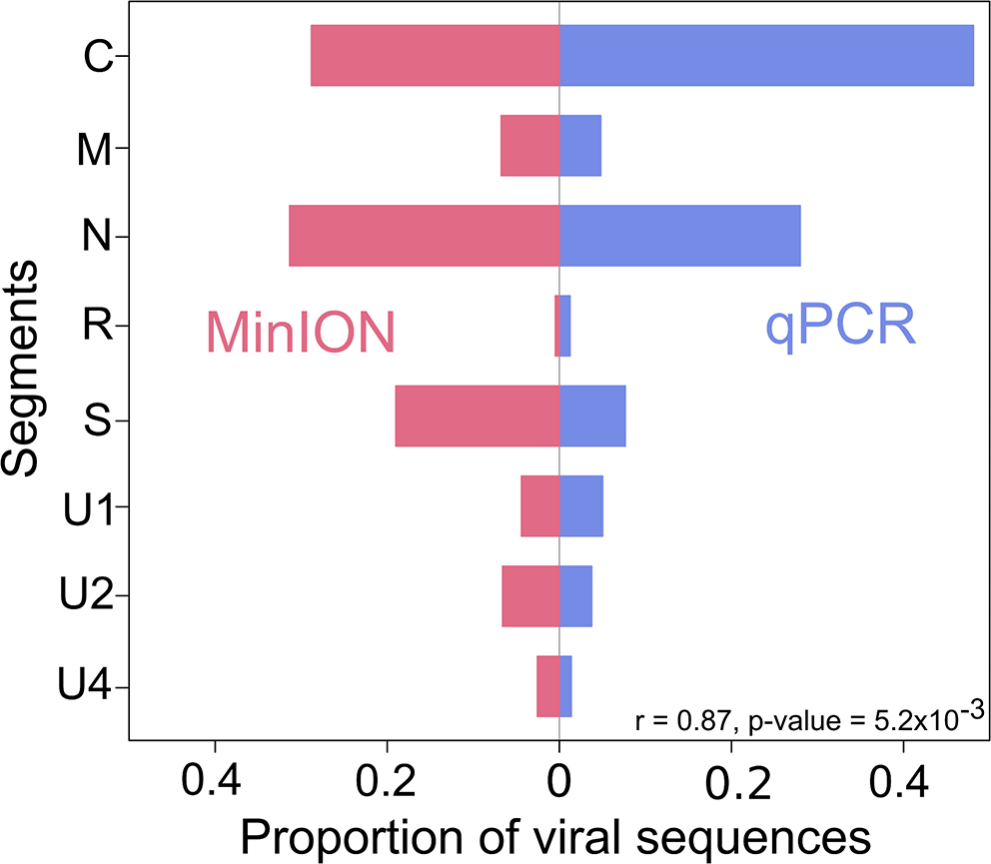
Comparison of FBNSV relative component frequencies as obtained by qPCR (left) and MinION sequencing (right). The p-value and correlation coefficient (r) of the Pearson correlation test of the two series are indicated at the bottom right

In order to assess the accuracy of the genomic formula estimate (GF, i.e. the estimated relative frequencies of each component; [37]), the FBNSV counts of TRC sequences were compared to those obtained from quantitative PCR [34]. In the common vetch, the TRC frequency of FBNSV DNA segments ranged from 0.5 to 31%. From this sample, frequencies were estimated at 1.2 to 48% using qPCR (Fig. 5). Both estimates were highly congruent with a correlation coefficient of 0.87 (i.e. the correlation of pairs of estimates of segments frequencies, ranging from zero for no congruence, to one for complete congruence) and a regression p-value of 5.2 × 10^− 3^. A previous study [35] reported global congruence of GFs obtained using quantitative PCR methods or next generation sequencing counts (including MinION) in the context of segmented RNA viruses [35] but highlighted specific variations related to each methods. Another study [16] focusing on a nanovirus, closely related to FBNSV, demonstrated that after RCA amplification, counts were congruent between Illumina and Nanopore sequencing. Here, giving largely congruent FBNSV genomic formulas, we observed that reads counts obtained after RCA and Nanopore sequencing are comparable to qPCR. However, deviations were found for three segments (C, R and S; Fig. 5) that could be related to the RCA biases [36, 38]. Finally, it must be noted that for ACMV, we found frequencies of 0.83 and 0.17 for the DNA-A and DNA-B components respectively, which may be indicative of a DNA-A component four times more frequent than the DNA-B component, an estimate compatible with the disparity in number of clones we obtained using RCA-RFLP (29 and four clones respectively). While these findings highlight the potential of RCA-MinION sequencing for quantitative genome analysis, they should be interpreted with caution. Additional studies using a broader set of sample types will be required to fully assess the quantitative potential of this method.

## Concluding remarks

Using the RCA-MinION approach, we recovered the complete genome of two multipartite CRESS-DNA viruses with two and eight genomic components. We demonstrated a complete match between the entire genomes of the CRESS-DNA viruses obtained by RCA-MinION and Sanger sequencing. Previous studies highlighted limits in the accuracy of MinION assemblies, however our results indicate that using the latest ONT chemistry and exploiting the multimeric characteristics of RCA products, RCA-MinION sequencing is a genuine alternative to Sanger sequencing for the detection and characterization of CRESS-DNA viruses. Importantly, all genomic components were recovered, making this approach particularly useful for the study of multipartite viruses or co-infections. Interestingly, multiplexing twelve distinct samples per flongle, would result in overall analysis cost (< 30€), similar to that of RCA-RFLP. However, for a given component size, the cost of RCA-RFLP increases linearly with the number of components/viruses whereas it remains constant for the RCA-MinION procedure. This method has the potential to become a valuable tool for CRESS-DNA virus diagnostics for personnel possessing minimal bioinformatic data management and analysis skills. Our study also revealed further applications of Nanopore sequencing, including population composition of viruses infecting plants. Furthermore, taking TRC sequences obtained for components of similar size, we were able to demonstrate that the number of TRC sequences correlated with the actual frequencies of the genomic components present in the sample. Further experiments are required to validate the use of RCA-MinION sequencing as a tool for studying the composition of virus populations. However, initial results suggest new possibilities for the study of species, strains, and defective or recombinant frequencies within a single sample.

## Electronic supplementary material

Below is the link to the electronic supplementary material.

Supplementary Material 1: **Additional Table 1**. Frequency of each mutation uncovered in the DNA-A and DNA-B Sanger sequences found in the TRC sequences with a score quality greater than or equal to 30 and a length greater than or equal to 2700 nt

Supplementary Material 2: **Additional Fig. 1**. Density plots representing the distribution of the average read quality of raw reads (A, C) and cleaned reads assigned to viruses (B, D) according to reads length (log10 scale) for the cassava (A, B) and common vetch samples (C, D)

## Data Availability

MinION data are available at the NCBI Short read archive under the BioProject PRJNA1192512. The full genome sequences of the isolate obtained from Sanger sequencing are available on GenBank under the accession numbers PQ261058-PQ261070.

## Abbreviations

ACMV: African cassava mosaic virus
BR: Broad Range
CRESS-DNA: Circular Rep-Encoding Single-Stranded -DNA
dNTP: Deoxynucleoside triphosphates
dsDNA: double stranded DNA
DTT: Dithiothreitol
FBNSV: Faba bean necrotic stunt virus
GF: Genome formula
HLV: Homopolymer Length Variation
INDELS: Insertion/Deletion
ONT: Oxford Nanopore Technology
qPCR: Quantitative PCR
RCA: Rolling Circle Amplification
RFLP: Restriction Fragment Length Polymorphism
REn: Replication enhancer
TR: Tandem repeat
TrAP: Transcriptional activator protein
TRC: Tandem repeat
