# Supplementary figures and images for: Improvement of Nanopore sequencing provides access to high quality genomic data for multi-component CRESS-DNA plant viruses
Source: Virol J. 2025 Mar 18;22:78. doi: 10.1186/s12985-025-02694-x (PMC11917030; doi:10.1186/s12985-025-02694-x)

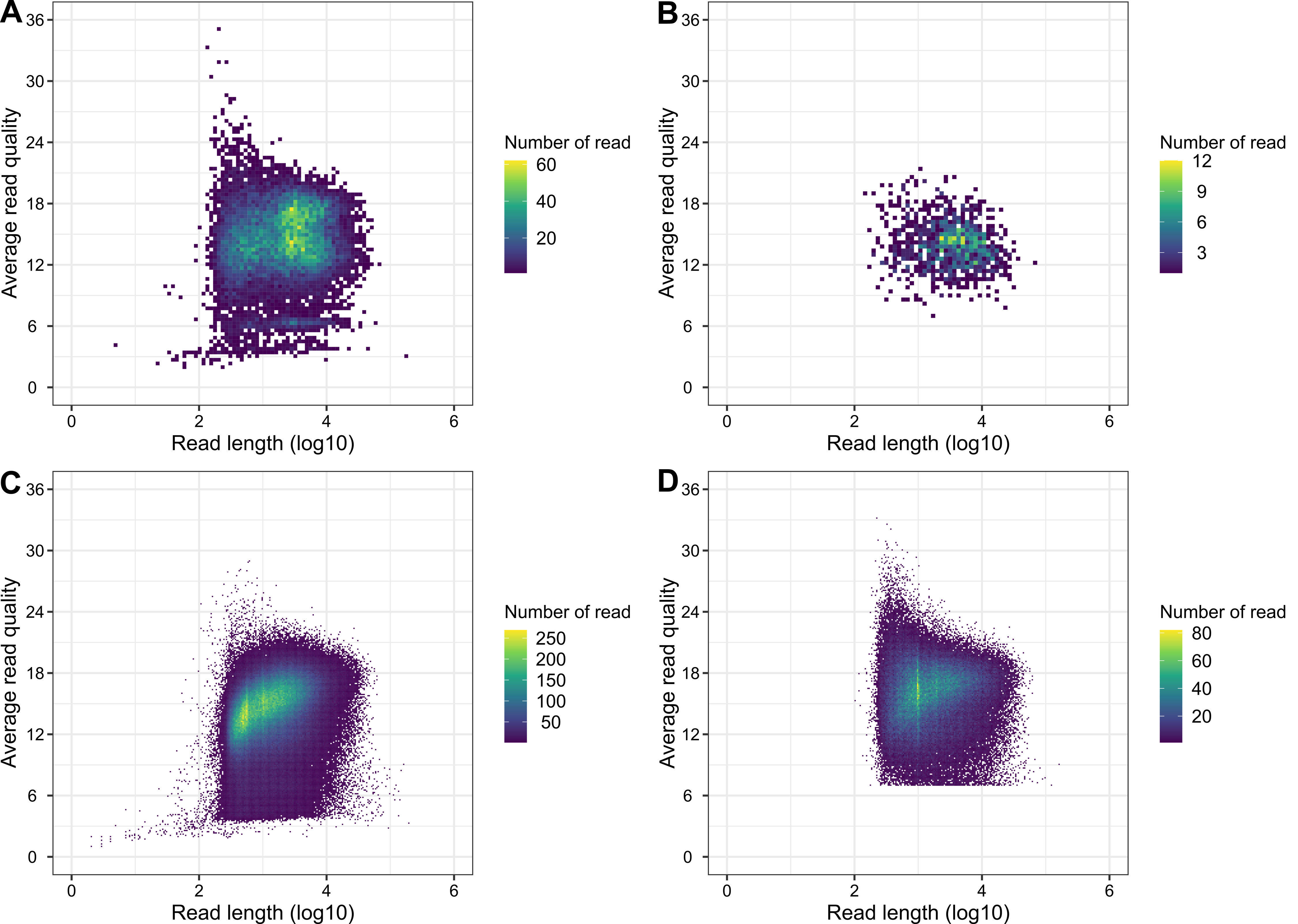

Supplement: Supplementary file 2 — Supplementary Material 2: Additional Fig. 1. Density plots representing the distribution of the average read quality of raw reads (A, C) and cleaned reads assigned to viruses (B, D) according to reads length (log10 scale) for the cassava (A, B) and common vetch samples (C, D) [file 12985_2025_2694_MOESM2_ESM.tif]
